# An Evaluation of Rapidly Progressive Dementia Culminating in a Diagnosis of Creutzfeldt–Jakob Disease

**DOI:** 10.1155/2018/2374179

**Published:** 2018-09-23

**Authors:** Parmvir Parmar, Curtis L. Cooper, Daniel Kobewka

**Affiliations:** ^1^Faculty of Medicine, University of Ottawa, Ottawa, ON, Canada; ^2^The Division of General Internal Medicine, Department of Medicine, University of Ottawa, Ottawa, ON, Canada; ^3^Ottawa Hospital Research Institute, Ottawa, ON, Canada; ^4^The Division of Infectious Diseases, Department of Medicine, University of Ottawa, Ottawa, ON, Canada

## Abstract

Rapidly progressive dementia is a curious and elusive clinical description of a pattern of cognitive deficits that progresses faster than typical dementia syndromes. The differential diagnosis and clinical workup for rapidly progressive dementia are quite extensive and involve searching for infectious, inflammatory, autoimmune, neoplastic, metabolic, and neurodegenerative causes. We present the case of a previously highly functional 76-year-old individual who presented with a 6-month history of rapidly progressive dementia. His most prominent symptoms were cognitive impairment, aphasia, visual hallucinations, and ataxia. Following an extensive battery of tests in hospital, the differential diagnosis remained probable CJD versus autoimmune encephalitis. He clinically deteriorated and progressed to akinetic mutism and myoclonus. He passed away 8 weeks after his initial presentation to hospital, and an autopsy confirmed a diagnosis of sporadic CJD. We use this illustrative case as a framework to discuss the clinical and diagnostic considerations in the workup for rapidly progressive dementia. We also discuss CJD and autoimmune encephalitis, the two main diagnostic possibilities in our patient, in more detail.

## 1. Case

A 76-year-old male linguistics professor was referred to the general internal medicine service by emergency medicine for a 2-week history of worsening confusion. He was independent for his activities of daily living (ADLs) and his instrumental activities of daily living (IADLs) at his baseline 6 months ago. The family endorsed a cognitive decline that started with memory issues, word-finding difficulty, and unsteady gait. They also endorsed a history of agitation and hallucinations at night. In the 2 weeks prior to his emergency room visit, his symptoms progressed at an even more rapid pace, with him being too weak to ambulate, and experiencing new incontinence of urine and stool. Until the worsening of his cognitive deficits, he was still working as a linguistics professor at the postsecondary level.

His past medical history was significant for coronary artery disease, hypertension, type 2 diabetes, asthma, and benign prostatic hyperplasia. There was no personal or family history of malignancy or dementia. He had never been screened for malignancy. There was no history of hunting or consuming game meat. His medications included ASA, candesartan, hydrochlorothiazide, metformin, glimepiride, iron supplements, multivitamins, and timolol eye drops. There were no over-the-counter medications, illicit drugs, or alcohol. On exam, his vitals were stable. His mucous membranes were dry, and his JVP was flat. His cardiac, respiratory, and abdominal exams were unremarkable. His neurological exam revealed a slight upward gaze palsy and velocity-dependent hypertonia in the upper extremities. There were no fasciculations or myoclonus. Reflexes and sensation were intact.

His white blood cell count was 2.7 × 10^9^ (normal 3.5–10.5), his hemoglobin was 134 g/L, and his platelets were 196 × 10^9^. The electrolytes and extended electrolytes were within normal limits aside from sodium of 125 mmol/L (normal 136–145). LTFs and bilirubin were within normal limits, and TSH was 2.35 (normal), and B12 level was 278 pmol/L (normal). Syphilis and HIV serologies were both negative, as was the antinuclear antibody (ANA). A diffusion-weighted MRI demonstrated diffuse parenchymal volume loss that was prominent for age and mild microangiopathic changes. His EEG was abnormal but nonspecific with irregular periods of 6–7 Hz theta activity, intermingled with short 2–4 Hz delta rhythms most prominent in the frontal regions. There was no alpha activity or obvious epileptiform, focal, or lateralizing features. CSF showed a nucleated cell count of 6 (normal 0–5), normal glucose, and slightly elevated protein at 0.55 g/L (normal 0.15–0.45 g/L). Oligoclonal bands were not detected in the CSF. CSF was negative for tau and 14-3-3 protein but positive for end-point quaking-induced conversion (EP-QuIC) at the National Microbiology Laboratory in Winnipeg. The paraneoplastic (anti-hu, ri, yo, ma2, cv2, and amphiphysin) antibody panel (mitogen) was negative.

He developed myoclonus and mutism, and he was discharged to a palliative care facility. He passed away 8 weeks after his initial emergency room presentation. The postmortem autopsy of the brain demonstrated microspongiosis, neuronal loss, and gliosis in the cortex, hippocampus, basal ganglia, and cerebellum, consistent with sCJD.

## 2. Rapidly Progressive Dementia

There is no currently accepted case definition for what constitutes a rapidly progressive dementia. Some authors suggest that a dementia that manifests and progresses within 2 years should be considered to have a rapidly progressing course [1], whereas others contend that cognitive deficits that follow a faster time course than typical Alzheimer's or vascular dementia should raise suspicion for a rapid dementia syndrome [2–4]. The differential diagnosis for rapidly progressive dementia is quite extensive and consists of infectious, inflammatory, autoimmune, neoplastic, metabolic, and neurodegenerative disease etiologies.

The clinical evaluation of a suspected rapidly progressive dementia syndrome should begin with a thorough patient history focusing on elucidating the first neurologic symptoms and establishing an accurate time course including new deficits [5–7].

Clinicians should also inquire about medications, especially anticholinergic medications and benzodiazepines as well as illicit drug use and alcohol consumption [6]. It is imperative to obtain a collateral history from friends and family, as well as a review of systems focusing on other affected organ systems [6]. The physical exam should focus on identifying autonomic dysfunction, extrapyramidal signs, fasciculations, and myoclonus and identifying stigmata of metabolic and neoplastic disease [1, 6].

There is a plethora of diagnostic tests that can be included in the workup of a rapidly progressive dementia syndrome. Selection and timing of ancillary tests should be done in a judicious, step-wise manner. Delirium and infectious and metabolic encephalopathies should be the targets of the initial investigations [2, 3]. The next layers of testing should search for autoimmune and neurodegenerative etiologies [2, 3]. Finally, testing can be extended to look for rare and uncommon presentations of disease entities, including atypical infections, depending upon elements in the patients' histories and exposures as well as abnormal results from the previous stages of investigation [2, 3].

Investigations should start with routine laboratory and imaging tests aimed at identifying common, reversible conditions [5, 8]. A complete blood count, electrolytes, extended electrolytes, B12, TSH, urinalysis, blood and urine cultures, a chest X-ray, and CT head should be ordered upfront in the evaluation of a patient with a possible rapidly progressive dementia to help distinguish dementia from delirium [5]. A lumbar puncture should be performed and CSF should be sent for cell count, bacteriology, and biochemical analysis to assess for meningitis [1, 5]. CSF should also be sent for 14-3-3, tau proteins, and EP-QuIC (end-point quaking test) to assess for CJD/prion disease [1, 3, 5]. An MRI brain with FLAIR sequence is useful to assess for autoimmune, neurodegenerative, and neoplastic causes of rapidly progressive dementia [1, 3]. Paraneoplastic and autoimmune antibody panels should also be tested [1]. An electroencephalogram (EEG) is useful to assess prion and neurodegenerative disease [1, 3, 5].

## 3. Creutzfeldt–Jakob Disease

Human prion diseases are quite uncommon with a worldwide incidence of 0.5–1 cases per million people [9, 10]. There exist genetic, acquired, and sporadic subtypes of prion disease [4, 9, 11, 12]. The genetic forms of prion disease are fatal familial insomnia and Gerstmann–Straussler–Scheinker disease [4, 9, 11, 12]. Acquired prion diseases include kuru, iatrogenic and variant CJD [4, 9, 13]. Sporadic CJD is the most common and accounts for 85–90% of all human prion diseases [10, 13, 14].

CJD can present with rapid cognitive decline, gait disturbance, and visual and behavioural disturbances and can progress to myoclonus and akinetic mutism [8, 10, 11, 13, 14]. CJD usually presents in the 6^th^ or 7^th^ decades of life; cases presenting before the age of 30 or after the age of 80 are exceedingly rare [14, 15]. CJD affects males and females equally [10, 14]. CJD has a rapidly progressing course and is uniformly fatal [15]. The median survival in sCJD is 5 months, with 90% of patients dying within 1 year [1, 4, 10, 14–16].

As CJD is relatively uncommon and unfamiliar to most clinicians, the diagnosis is difficult and CJD is often misdiagnosed. CJD is caused by prions which are pseudoinfectious, self-propagating proteinaceous particles that cause aggregation, spongiform changes, and neuronal loss [11, 12, 14, 15]. Definitive diagnosis of CJD is through histpathologic analysis of brain tissue obtained from brain biopsy or, more commonly, at autopsy [1, 7, 10, 12, 15]. Supportive diagnostic modalities that can support a diagnosis of CJD include EEG, MRI, CSF 14-3-3, and EP-QuIC. Early CJD may manifest as nonspecific slowing on EEG, whereas the characteristic triphashic periodic sharp wave complexes may present later in the disease course [3, 4, 11, 12, 15]. The sensitivity and specificity of EEG for detecting CJD is 50–66% and 74–91%, respectively [3, 12]. The pulvinar sign on MRI refers to bilateral FLAIR hyperintensities in the pulvinar and thalamic nuclei and can be seen in variant and sporadic CJD [7, 9, 10, 12]. MRI is 91% sensitive and 95% specific for CJD [3, 12, 15]. 14-3-3 and tau proteins in CSF are sensitive for neuronal injury although they are not specific for CJD [7, 12]. EP-QuIC is an empirically validated assay that utilizes the intrinsic properties of disease-associated prion protein in patients' CSF to cause misfolding and aggregation of the recombinant prion protein. The protein aggregates interact with a dye resulting in detectable changes in its fluorescence pattern [17]. EP-QuIC is 80–90% sensitive and 99–100% specific for the diagnosis of prion disease [4, 12].

A diagnosis of probable CJD requires rapidly progressive dementia, and two of four of myoclonus, visual or cerebellar symptoms, pyramidal/extrapyramidal symptoms, or akinetic mutism and a positive result on a supporting test (EEG, 14-3-3, or MRI) [14, 15]. EP-QuIC is a newer diagnostic test, and it is not yet included in the WHO diagnostic criteria for CJD [14, 15].

All subtypes of CJD are progressive and unequivocally result in death. Aside from treatments aimed at controlling symptoms, there are no effective therapies that stop or thwart the progression of disease [7]. Despite the limited avenues for treatment, establishing a diagnosis of prion disease is valuable. In most jurisdictions, CJD is a reportable disease that is surveyed by public health authorities [14]. Diagnoses of familial variants of the disease have implications for genetic testing for family members. With respect to providing direct patient care, once a diagnosis of CJD is made, a clear trajectory of illness is identified. Patients and their families should be referred to physicians trained in providing palliative care for assistance in transitioning to hospice care [14, 18].

## 4. Autoimmune Encephalitis

Autoimmune encephalitis is an important disease etiology that is included in the differential diagnosis of rapidly progressive dementia. The initial presentation of autoimmune encephalitis is highly variable and depends on the culprit antibody and associated disease entity. Psychiatric manifestations and cognitive decline are the most frequently observed initial symptoms in autoimmune encephalitis [3, 19]. There are several subgroups of autoimmune encephalitis: classic paraneoplastic encephalitis, diseases associated with autoantibodies against ion channels, diseases associated with autoantibodies against intracellular synaptic proteins, and finally, autoimmune encephalitis in which the antigens are not clearly defined [19].

Autoimmune antibodies against extracellular epitopes of ion channels are intrinsically pathologic; in addition to cognitive decline, they can also result in unique clinical manifestations [3, 19]. Anti-leucine-rich glioma-inactivated 1 (LGI1) limbic encephalitis is caused by antibodies against the LGI1 element of the voltage-gated potassium channel (VGKC) complex [20]. LGI1 encephalitis presents with seizures, dysautonomia, and hyponatremia secondary to SIADH [3, 6, 20, 21]. Overt psychosis and autonomic dysfunction are features of N-methyl-D-aspartate (NMDA) receptor encephalitis; it is associated with ovarian teratoma and testicular cancer [3].

Unlike autoimmune antibodies against ion channels, paraneoplastic antibodies are not in themselves pathologic. The neuropsychiatric symptoms including anxiety and hallucinations with a fluctuating course tend to be present in paraneoplastic encephalitis [1, 3, 19]. Paraneoplastic antibodies often precede the diagnosis of the underlying malignancy, sometimes by 1 year or more [2, 19].

GAD65 (glutamic acid decarboxylase) autoimmune encephalitis is an example of a disease associated with autoantibodies against an intracellular synaptic protein [3, 19]. GAD65 encephalitis presents with stiff person syndrome, a progressive rigidity, and myoclonus of the truncal muscles [3, 19]. Interestingly, GAD65 disease is also associated with treatment-resistant epilepsy and new onset of type 1 diabetes [3, 19].

Contrary to CJD, which typically has bland CSF biochemistry, CSF in autoimmune encephalitis usually demonstrates a lymphocytic pleocytosis, with elevated protein, and the occasional presence of oligoclonal bands [3]. EEG and MRI are much less useful in establishing a diagnosis of autoimmune encephalitis as compared with CJD. MRI is frequently normal in cases of autoimmune encephalitis and cannot exclude this diagnosis [19]. EEG is useful for monitoring seizure activity associated with some forms of autoimmune encephalitis; specific EEG findings may be observed in NMDAR encephalitis and LGI1 (leucine-rich glioma-inactivated) limbic encephalitis [19]. Histopathology is neither practical nor specific for autoimmune encephalitis [19].

Autoimmune encephalitis remains an important diagnostic consideration in the evaluation of rapidly progressive dementia, as it is treatable and potentially reversible. Most autoimmune encephalitis tends to be responsive to steroids, typically prescribed as solumedrol 1 g IV daily for 3–5 days followed by a taper [1, 3, 6]. Plasma exchange therapy and intravenous immunoglobulin (IVIG) can also be considered, primarily for confirmed or suspected autoimmune encephalitis that does not respond to steroids alone [1, 3, 6, 19]. Second-line therapy for treatment of autoimmune encephalitis includes rituximab and cyclophosphamide [3, 19]. Of note, paraneoplastic encephalitis typically does not respond to steroids or plex, but may improve with treatment of the associated malignancy [3, 19].

## 5. Conclusion

Rapidly progressing dementia is an interesting clinical scenario with a multitude of diagnostic possibilities. It requires a thorough clinical assessment and diagnostic workup, which should focus on finding reversible, treatable conditions. CJD and autoimmune encephalitis are included in the differential diagnosis of rapidly progressive dementia. These two disease entities can present somewhat similarly; however, they differ dramatically in their clinical trajectory and prognosis. Autoimmune encephalitis presents with neuropsychiatric symptoms, and it is highly responsive to treatment with steroids, whereas CJD presents with cognitive decline, extrapyramidal symptoms and gait disturbance, and myoclonus; it is uniformly fatal, usually within 12 months of onset of symptoms.
